# Tumour necrosis factor alpha-induced protein 3-interacting protein 3 overexpression protects against arrhythmogenic remodelling in the heart failure mice

**DOI:** 10.1093/europace/euaf002

**Published:** 2025-01-13

**Authors:** Hongjie Yang, Xiaoyan Shen, Huibo Wang, Wei Shuai

**Affiliations:** Department of Cardiology, Renmin Hospital of Wuhan University, No. 238 Jiefang Road, Wuhan 430060, Hubei, P.R. of China; Department of Anesthesiology, Renmin Hospital of Wuhan University, No. 238 Jiefang Road, Wuhan 430060, Hubei, P.R. of China; Department of Cardiology, The First College of Clinical Medical Science, China Three Gorges University & Yichang Central People's Hospital; Institute of Cardiovascular Diseases, China Three Gorges University, Hu Bei Clinical Research Center for Ischemic Cardiovascular Disease, No. 183 Yiling Avenue, Yichang 443000, Hubei, China; Department of Cardiology, Renmin Hospital of Wuhan University, No. 238 Jiefang Road, Wuhan 430060, Hubei, P.R. of China

**Keywords:** Isoproterenol, Heart failure, Ventricular arrhythmia, Inflammation

## Abstract

**Aims:**

Ventricular arrhythmias (VAs), which can lead to sudden cardiac death, are the primary cause of mortality in patients with heart failure (HF). However, the precise mechanisms underlying these arrhythmias are not well understood. Recent studies have implicated tumour necrosis factor alpha-induced protein 3-interacting protein 3 (TNIP3) in pathological cardiac hypertrophy. Nevertheless, its role in isoproterenol (ISO)-associated VAs remains elusive.

**Methods and results:**

We overexpressed TNIP3 in the myocardium using an adeno-associated virus 9 system, administered via tail vein injection. C57BL/6 mice received daily subcutaneous injections of ISO for two consecutive weeks to establish an HF model. We performed histopathology and electrophysiological studies to assess ventricular structural remodelling, electrical remodelling, and susceptibility to VAs. Additionally, RNA sequencing (RNA-Seq) and western blot analysis were conducted to elucidate the underlying mechanisms. The expression of TNIP3 was up-regulated following ISO treatment. TNIP3 overexpression significantly reversed ISO-induced cardiac dysfunction, fibrosis, electrical remodelling, and VAs susceptibility. Accordingly, RNA-Seq identifies that the inflammatory response takes an important role in ISO-induced Vas, and TNIP3 overexpression could alleviate ISO-induced cardiac proinflammatory response by promoting M1 to M2 macrophage polarization. Mechanistically, PI3K/Akt/NF-κB signalling is responsible for the protective effect of TNIP3 overexpression on ISO-induced HF. And PI3K/Akt signalling activation offset the protective effect of TNIP3 overexpression on ISO-induced cardiac inflammation and VAs.

**Conclusion:**

The findings of this study highlight the critical role of TNIP3 in ISO-associated cardiac remodelling and VAs, which are induced by the inhibited activation of the PI3K/Akt/NF-κB signalling pathway.

## Introduction

Heart failure (HF) is the clinical manifestation of all cardiovascular diseases at the end stage.^1,2^ Due to the increase of its incidence rate and mortality, HF has become a noticeable clinical issue, affecting more than 6 million people in the United State.^3^ Though much progress has been made in the treatment of HF, certain patients continue to have HF symptoms that are resistant to the current range of guidelines-based treatments; the mortality rate of HF patients remains high.^4,5^ It was reported that the 5-year survival rate of HF patients is less than 50%, and sudden cardiac death is the most common cause of death in HF patients, approximately half of HF patients die from sudden cardiac death caused by ventricular arrhythmia (VA).^6,7^ Hence, it is critically important to explore novel strategies to inhibit the HF-induced VAs.

Up to now, the precise mechanism of HF-associated VAs has not been fully elucidated, cardiac remodelling including structural remodelling and electrical remodelling, is considered a key mechanism by which HF triggers VAs.^8,9^ Structural remodelling, such as myocardial hypertrophy, fibrosis, inflammation, oxidative stress, etc., can lead to a decrease in ventricular electrical conduction velocity (CV) and uneven conduction, providing a substrate for electrical re-entry and increasing susceptibility to VAs. The molecular basis of electrical remodelling mainly includes abnormal calcium handling and abnormal sodium and potassium ion channels in cardiomyocytes, leading to ventricular depolarization and triggering activity, thereby causing VAs.^10,11^ Therefore, exploring new targets for intervening in cardiac remodelling is expected to provide new treatment strategies for HF-induced VA.

Tumour necrosis factor alpha-induced protein 3-interacting protein 3 (TNIP3), also known as ABIN3, is a protein that has been linked to inflammatory bowel disease,^12^ which has been demonstrated to adversely control NF-κB activation in response to lipopolysaccharide (LPS).^13^ According to earlier research, TNIP3 could inhibit TAK1 activation to function as a unique endogenous suppressor of non-alcoholic steatohepatitis.^14^ Moreover, by degrading LATS2, TNIP3 overexpression has demonstrated protective benefits against hepatic ischaemia-reperfusion injury.^15^ Recently, Shi et al. checked the role of TNIP3 in transverse aortic constriction (TAC)-induced pathological cardiac hypertrophy and found that TNIP3 serves as a novel suppressor of pathological cardiac hypertrophy by promoting STAT1 stability.^16^ However, the potential role of TNIP3 in VA induced by isoproterenol (ISO)-associated HF has not been explored. Hence, the present study intended to check the role of TNIP3 in HF-induced VA and the potential underlying mechanisms.

## Methods

The extended methods section describes all the procedures and protocols, which can be found in the Supplementary material online, *Materials*.

### Animals and treatment

Male C57BL/6 mice (8- to 10-week-old) were purchased from the Chinese Academy of Medical Sciences and performed in accordance with the Guidelines for the Care and Use of Laboratory Animals published by the United States National Institutes of Health (NIH Publication, revised 2011). The use of animals was reviewed and approved by the Ethics Committee of the Renmin Hospital of Wuhan University. Mice were allowed 1 week to acclimatize to a stable environment before experiments began. Mice were individually housed in plastic cages with bedding, ad libitum food, and tap water. The cages were maintained at 22 ± 2°C and a 12:12-h light/dark cycle. C57BL/6 mice were randomly divided into four groups: CTL + AAV9-GFP, CTL + AAV9-TNIP3, ISO + AAV9-GFP, and ISO + AAV9-TNIP3. To overexpress TNIP3 in mice, the mice were injected via the tail vein with adeno-associated viral 9 vector (AAV9) (Genechem Co., Ltd, Shanghai, China) carrying TNIP3 (AAV9-cTNT-TNIP3) or AAV9 vector that expressed green fluorescent protein (GFP) as control (AAV9-GFP). AAV9-TNIP3 or AAV9-GFP was intramyocardially injected with 2 × 10^11^ viral genome particles per mouse. Following 4 weeks of AAV9-cTNT-TNIP3 or AAV9-cTNT-GFP injection. Chronic ISO infusion was used to establish an HF model, mice were injected subcutaneously with ISO (30 mg/kg/day, Sigma-Aldrich, MO, USA) or with 0.9% saline daily for 2 weeks as described previously.^17^ SC79, a PI3K agonist, was treated (10 mg/kg, intraperitoneal injection, MCE, HY-18749) at 0.5 h before the ISO infusion.^18^ All the mice were monitored and weighed weekly and were sacrificed following a 2-week ISO-induced HF mice model, with hearts collected for further experiment.

### Statistical analysis

Statistical analyses were conducted using SPSS 24 and GraphPad Prism 9.0. Continuous variables are expressed as means ± SEM. The sample size for each analysis is detailed in the figure legends. For data following a Gaussian distribution, comparisons between two groups were made using either an unpaired Student's *t*-test (assuming equal variances) or an unpaired Student's *t*-test with Welch’s correction (for unequal variances). Multiple group comparisons were carried out using either one-way or two-way analysis of variance, as deemed appropriate. *Post hoc* multiple comparison tests were conducted using the Tukey test. For non-Gaussian data, non-parametric analyses were performed, using the Mann–Whitney *U* test for two-group comparisons and the Kruskal–Wallis test for comparisons among multiple groups. A *P*-value <0.05 was considered statistically significant.

## Results

### Tumour necrosis factor alpha-induced protein 3-interacting protein 3 was up-regulated in isoproterenol-induced cardiomyocytes

**Figure 1:**
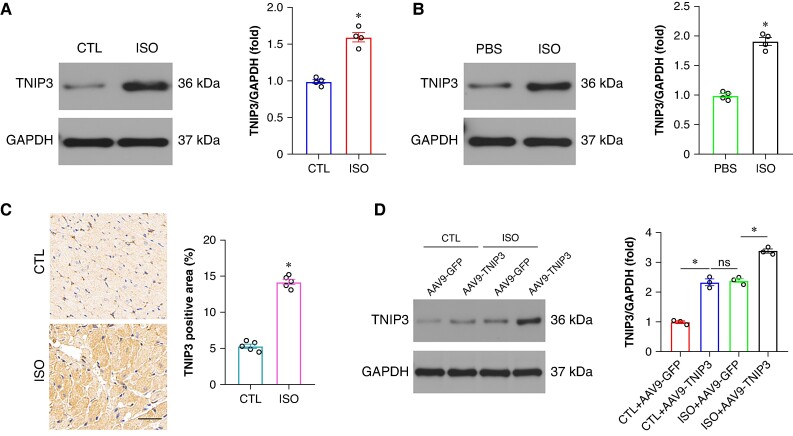
TNIP3 was up-regulated in ISO-induced cardiomyocytes. (*A*) Representative western blot and statistical results of TNIP3 protein expression *in vivo* (*n* = 4). (*B*) Representative western blot and statistical results of TNIP3 protein expression *in vitro* (*n* = 4). (*C*) Immunohistochemical staining of TNIP3 in heart tissues from control and ISO mice (*n* = 5). (*D*) Representative western blot and statistical results of TNIP3 protein expression following TNIP3 overexpression *in vivo* (*n* = 4). *P < 0.05. ISO, isoproterenol; TNIP3, tumour necrosis factor alpha-induced protein 3-interacting protein 3.

To explore the role of TNIP3 in ISO-induced VAs, we first checked the expression of TNIP3 in ISO-induced cardiomyocytes. Western blot analysis revealed an elevated TNIP3 protein expression in ISO-induced cardiomyocyte both in mice and in H9c2 cells (*Figure* *1A* and *B*). The up-regulated TNIP3 expression was further verified by immunohistochemistry of ventricular myocardium in ISO-induced hearts (*Figure 1C*). The increased TNIP3 expression both *in vivo* and *in vitro* suggested that TNIP3 might be involved in the pathological process in ISO-induced cardiomyocytes.

### Tumour necrosis factor alpha-induced protein 3-interacting protein 3 overexpression improved cardiac function and fibrosis in isoproterenol-induced heart failure mice

**Figure 2:**
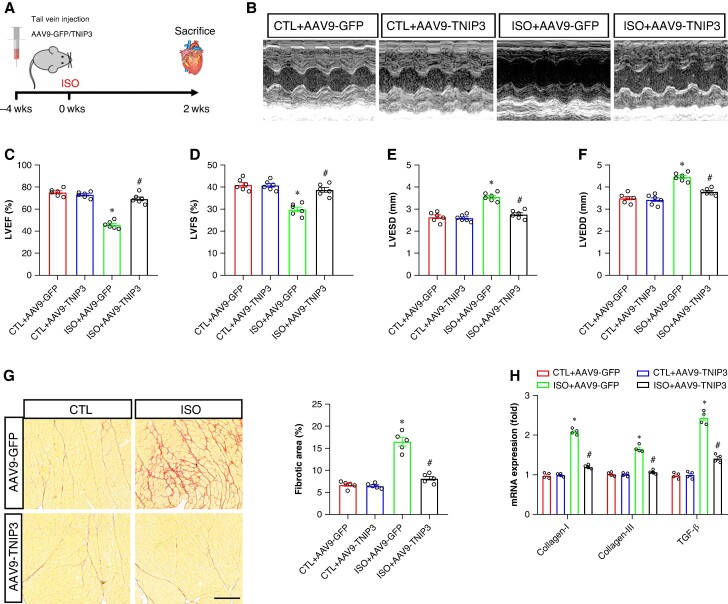
TNIP3 overexpression improved cardiac function and fibrosis in ISO-induced heart failure mice. (*A*) Schematic diagram of experimental procedure *in vivo* study. (*B*) Representative echocardiogram images. **(***C–D*) Quantitative of LVEF and LVFS (*n* = 6). (*E–F*) Quantitative of LVESD and LVEDD (*n* = 6). (*G*) Representative images of PSR-stained heart sections and quantitative of percentage of the fibrotic area (*n* = 5 per group). (*G*) Quantitative of mRNA levels of *collagen-I*, *collagen-III*, and *TGF-β* (*n* = 4 per group). *P < 0.05 vs. CTL + AAV9-GFP group. #*P* < 0.05 vs. ISO + AAV9-GFP group. ISO, isoproterenol; LVEDD, left ventricular end-systolic diameter; LVEF, left ventricular ejection fraction; LVESD, left ventricular end-diastolic diameter; LVFS, left ventricular fractional shortening; PSR, Picric-Sirius red.

To address the function of TNIP3 in failing hearts. AAV9-GFP and AAV9-TNIP3 were delivered into failing mouse models via tail vein injection 4 weeks before ISO injection (*Figure 2A*). And the transfection efficiency of AAV9-GFP and AAV9-TNIP3 was shown in *Figure 1D*, AAV9-TNIP3 significantly increased TNIP3 protein compared to AAV9-GFP, and ISO further increased TNIP3 protein expression. After 2 weeks of ISO injection, echocardiography was performed. ISO injection significantly worsens cardiac function and ventricular enlargement, reflected by decreased left ventricular ejection fraction (LVEF) and left ventricular fractional shortening (LVFS) and increased left ventricular end-diastolic diameter (LVEDD) and left ventricular end-systolic diameter (LVESD) compared with the CTL+ AAV9-GFP group (*Figure 2B–F*). Notably, ISO-induced cardiac dysfunction and ventricular enlargement were significantly reversed by TNIP3 overexpression (*Figure 2B–F*). Cardiac fibrosis is a hallmark mechanism of HF-induced VAs. We then examined the effect of TNIP3 overexpression on ISO-induced cardiac fibrosis. Picric-Sirius red (PSR) staining and RT–PCR were used to assess cardiac fibrosis. As shown in *Figure 2G*, increased cardiac fibrosis was found in ISO-induced heart compared with CTL group. The result was further verified by significantly mRNA expression of fibrotic indicators, including collagen-I, collagen-III, and TGF-β (*Figure 2H*). All these alterations induced by ISO were reversed by TNIP3 overexpression, which showed that TNIP3 overexpression mitigated ISO-induced cardiac dysfunction and cardiac function.

### Tumour necrosis factor alpha-induced protein 3-interacting protein 3 overexpression diminished ventricular arrhythmia susceptibility in isoproterenol-induced heart failure mice

**Figure 3:**
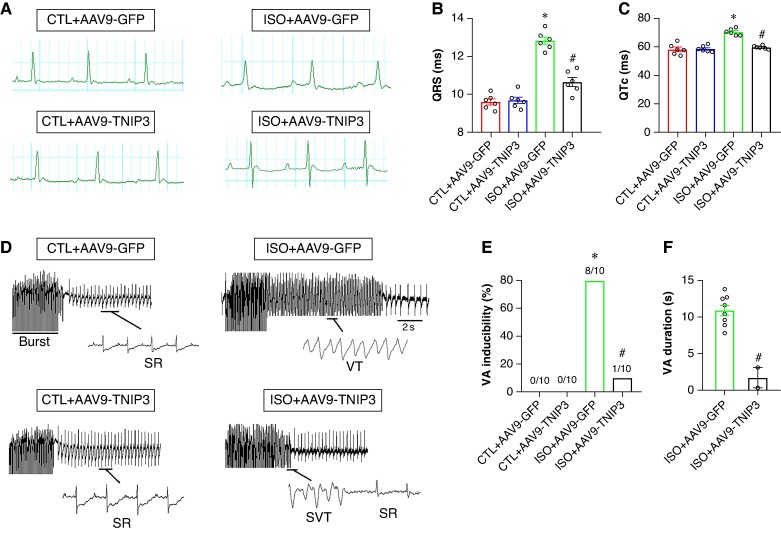
TNIP3 overexpression diminished ventricular arrhythmia susceptibility in ISO-induced heart failure mice. (*A*) Representative electrocardiogram images. (*B–C*) Quantitative of QRS interval and QTc (*n* = 6 per group). (*D*) Representative examples of VA induced by burst-stimulating. (*E*) Ratio of burst-induced VAs and (*F*) duration of VA (*n* = 10 per group). *P < 0.05 vs. CTL + AAV9-GFP group. #*P* < 0.05 vs. ISO + AAV9-GFP group. SR, sinus rhythm; VA, ventricular arrhythmia.

To determine factors that are associated with the susceptibility for arrhythmias. The surface ECG indicated the effects of TNIP3 overexpression on cardiac electrical activity. As shown in *Figure 3A*, after 2 weeks of ISO treatment, compared with the CTL group, ISO treatment displayed prolonged QRS duration and QTc (*Figure* *3B* and *C*). The alterations of ECG parameters induced by ISO were normalized following TNIP3 overexpression (*Figure* *3B* and *C*).

Burst pacing was used to detect the effect of TNIP3 overexpression on VAs vulnerability induced by ISO. VAs could not be induced in the CTL group and CTL + AAV9-GFP group (*Figure 3D*). The VAs induction ratio in the ISO group was significantly increased compared with the CTL group (80% vs. 0%, *P* < 0.01, *Figure* *3D* and *E*). TNIP3 overexpression markedly decreased VAs susceptibility compared with the ISO group (10% vs. 80%, *P* < 0.01, *Figure* *3D* and *E*). Moreover, TNIP3 overexpression significantly decreased the duration of VAs compared with the ISO group (*Figure 3F*). These data showed that ISO increased VAs inducibility and TNIP3 overexpression decreased ISO-induced VAs.

### Tumour necrosis factor alpha-induced protein 3-interacting protein 3 overexpression reversed conduction velocity reduction in isoproterenol-induced heart failure mice

**Figure 4:**
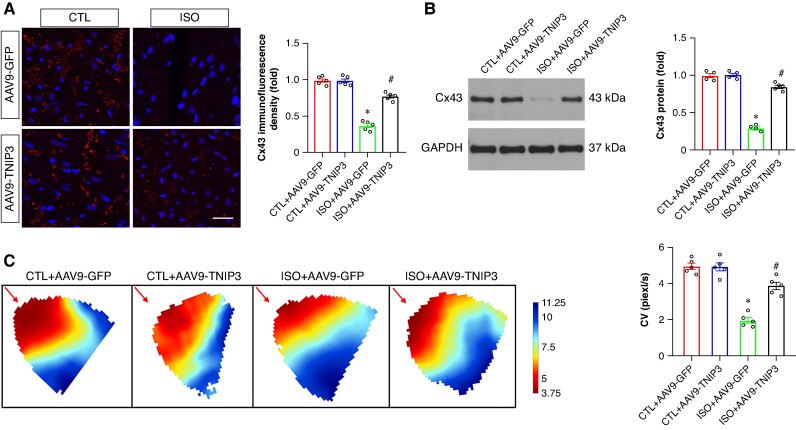
TNIP3 overexpression reversed CV reduction in ISO-induced heart failure mice. (*A*) Typical examples and quantitative results of Cx43 immunofluorescence staining (*n* = 5). (*B*) Representative western blot and statistical results of Cx43 protein expression (*n* = 4). (*C*) Representative image of epicardial conduction recorded by optical mapping and quantitative results of CV (*n* = 5). *P < 0.05 vs. CTL + AAV9-GFP group. #*P* < 0.05 vs. ISO + AAV9-GFP group. CV, conduction velocity. Cx43, Connexin 43.

Connexin 43 (Cx43) is involved in cell-to-cell communication regulation that affects cardiac myocyte electrical coupling. The incidence of VAs is closely associated with the decreased Cx43 in the ventricle. As exhibited in *Figure 4A*, decreased Cx43 immunofluorescence density was found in ISO group compared with the CTL group, and TNIP3 overexpression markedly increased Cx43 immunofluorescence density in the ventricle compared with the ISO group (*Figure 4A*). Moreover, we tested Cx43 protein expression in the ventricle, and the results found decreased Cx43 protein expression in the ISO group compared with the CTL group. And TNIP3 overexpression significantly increased Cx43 protein expression (*Figure 4B*).

Conduction velocity in the ventricle can be altered by Cx43 reduction. We then evaluated the effect of TNIP3 overexpression on CV by performing optical mapping. Representative activation map was exhibited in *Figure 4C*. The CV in the ISO group was significantly decreased compared with the CTL group (*Figure 4C*), and TNIP3 overexpression significantly increased ventricle CV compared with the ISO group (*Figure 4C*).

### Tumour necrosis factor alpha-induced protein 3-interacting protein 3 overexpression reversed electrical remodelling in isoproterenol-induced heart failure mice

**Figure 5:**
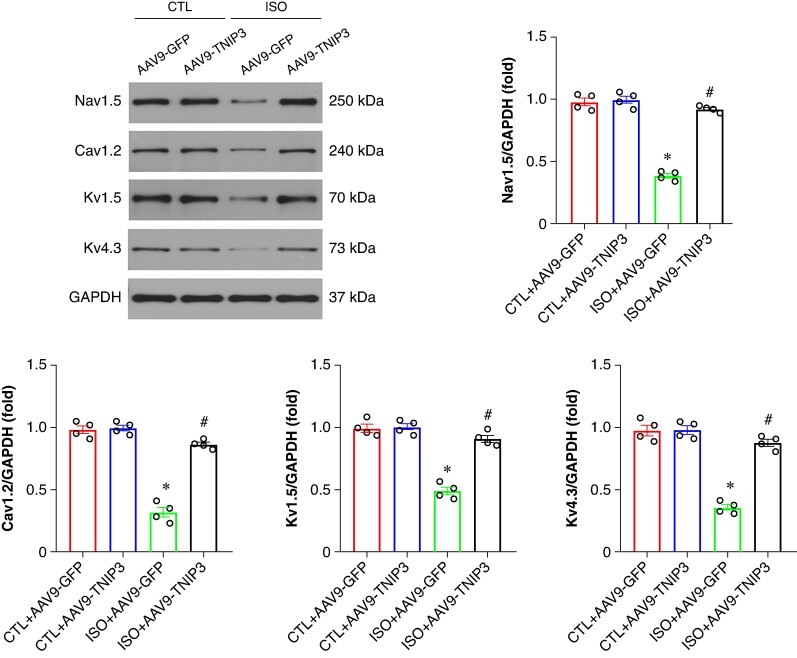
TNIP3 overexpression reversed electrical remodelling in ISO-induced heart failure mice. (*A–E*) Representative western blot and statistical results of Cav1.2, Kv1.5, and Kv4.3 protein expression (*n* = 4). *P < 0.05 vs. CTL + AAV9-GFP group. #*P* < 0.05 vs. ISO + AAV9-GFP group.

Ventricular electrical remodelling is a hallmark pathophysiological mechanism of VA. Alterations in ion channel protein, including voltage-gated L-type calcium channel (Cav1.2) and potassium voltage-gated channel (Kv1.5 and Kv4.3), play a critical role in the arrhythmogenic mechanisms underlying the initiation of HF-induced VA. We then examined the protein expression of Cav1.2, Kv1.5, and Kv4.3 in ventricle. Western blot analysis revealed decreased Cav1.2, Kv1.5, and Kv4.3 protein expression in ISO hearts compared with the CTL hearts, TNIP3 overexpression significantly increased protein expression of Cav1.2, Kv1.5, and Kv4.3 protein expression compared with the ISO group (*Figure 5*). Altogether, these data demonstrated that TNIP3 overexpression significantly alleviated ISO-induced ventricular electrical remodelling.

### Tumour necrosis factor alpha-induced protein 3-interacting protein 3 overexpression promoted M2 macrophage polarization in isoproterenol-induced heart failure hearts

**Figure 6:**
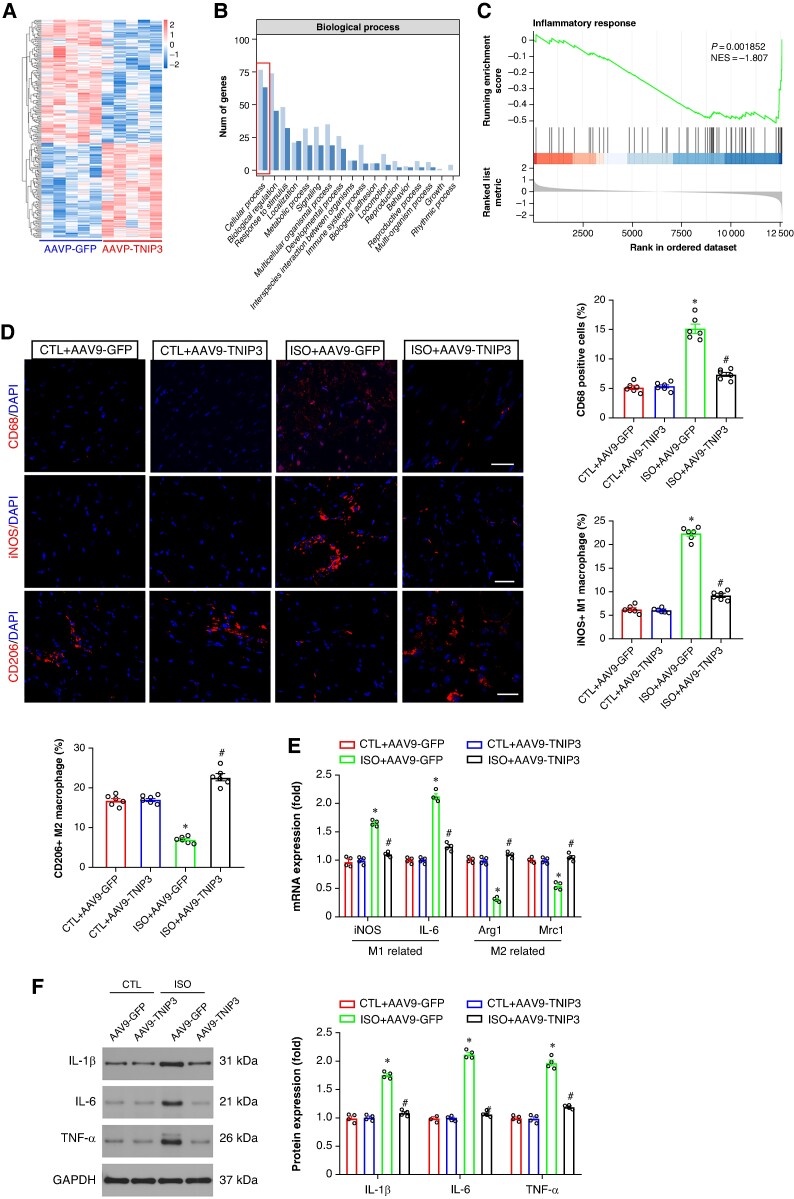
TNIP3 overexpression alleviated inflammatory response in ISO-induced heart failure mice. (*A*) Heatmap of RNA-Seq expression levels of DEGS in ISO + AAV9-GFP vs. ISO + AAV9-TNIP3 ventricles (*n* = 5 per group). (*B*) Gene Ontology (GO) enrichment analysis of the biological process. (*C*) Gene set enrichment analysis (GSEA). (*D*) Representative CD68, iNOS, and CD206 immunofluorescence and quantitative results of CD68, iNOS, and CD206-positive cells (*n* = 6 per group). (*E*) Quantitative results of iNOS, IL-6, Arg1, and Mrc1 mRNA expression (*n* = 4 per group). (*F*) Representative western blot and statistical results of protein levels of IL-1β, IL-6, and TNF-α (*n* = 4 per group). *P < 0.05 vs. CTL + AAV9-GFP group. #*P* < 0.05 vs. ISO + AAV9-GFP group. DEGs, differential expression genes; IL-1β, interleukin 1β; IL-6, interleukin 6; RNA-Seq, RNA sequencing; TNF-α, tumour necrosis factor α.

To clarify the potential mechanisms underlying the ISO-induced VA, RNA sequencing (RNA-Seq) was performed in ISO + AAV9-GFP and ISO + AAV9-TNIP3 groups. The heat map indicated the differentially expressed genes (DEGs; *Figure 6A*). After 2 weeks of ISO injection, 108 genes were significantly up-regulated, while 140 genes were down-regulated in ISO + AAV9-TNIP3 group compared with the ISO + AAV9-GFP group. Gene Ontology (GO) enrichment analysis and gene set enrichment analysis (GSEA) showed significant biological processes, and inflammatory response was the most altered biological process before and after TNIP3 overexpression, and TNIP3 overexpression significantly suppressed ISO-induced inflammatory response (*Figure* *6B* and *C*). Recent evidence showed that the elevation of infiltrated macrophages and proinflammatory mediators is observed in ISO-induced cardiac remodelling.^19,20^ To verify the findings in RNA-Seq and clarify the effect of TNIP3 overexpression on macrophage infiltration and M1 to M2 macrophage polarization transition, immunofluorescence was performed to assess macrophage infiltration by quantifying the percentage of CD68-positive macrophages in the hearts; meanwhile, to identify the subtype of infiltrated macrophages in the heart, the markers for M1 [inducible nitric oxide synthase (iNOS) immunofluorescence, iNOS, and IL-6 mRNA] and M2 (CD206 immunofluorescence, Arg1 and Mrc1 mRNA) were examined. The results indicated that ISO significantly increased CD68 immunofluorescence intensity, and TNIP3 overexpression markedly decreased CD68 immunofluorescence intensity (*Figure 6D*). The number of M1 macrophages (iNOS+) in ISO-treated heart was significantly increased compared with CTL heart and TNIP3 overexpression significantly decreased iNOS + macrophage numbers, suggesting that TNIP3 overexpression suppresses M1 macrophage polarization and reduces the infiltrated M1 macrophages in the hearts, further qRT–PCR revealed that TNIP3 overexpression inhibited mRNA expression of M1 macrophage markers iNOS and IL-6 (*Figure 6E*). Moreover, TNIP3 overexpression significantly increased CD206+ (M2 macrophage markers) macrophages numbers compared to ISO-treated hearts (*Figure 6D*). TNIP3 overexpression also significantly up-regulated the expression of M2-associated genes, including Arg1 and Mrc (*Figure 6E*). And the result of western blot analysis indicated that ISO treatment significantly increased proinflammatory cytokines IL-1β, IL-6, and TNF-α protein expression compared to CTL hearts, and TNIP3 overexpression significantly inhibited ISO-induced proinflammatory cytokines (*Figure 6F*). Altogether, these clues indicated that TNIP3 overexpression partially inhibited proinflammatory response by promoting M1 to M2 macrophage polarization induced by ISO treatment.

### PI3K/Akt/NF-κb signalling is responsible for the protective effect of tumour necrosis factor alpha-induced protein 3-interacting protein 3 overexpression on isoproterenol-induced heart failure

**Figure 7:**
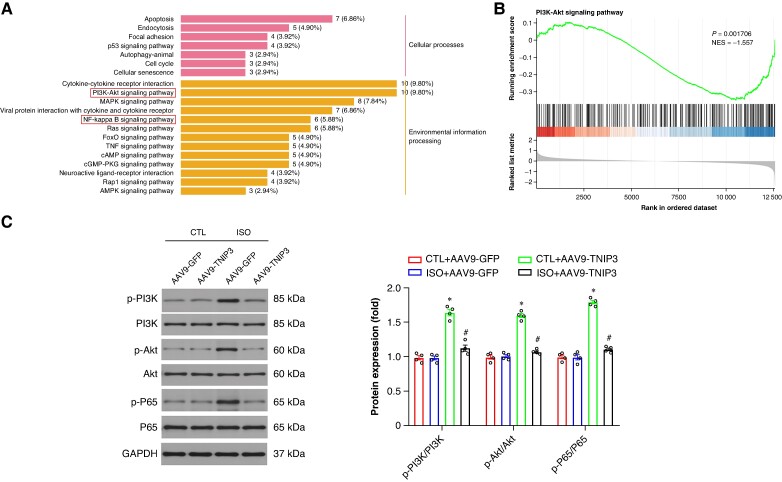
PI3K/Akt/NF-κB signalling is responsible for the protective effect of TNIP3 overexpression on ISO-induced heart failure. (*A*) KEGG analysis. (*B*) GSEA of KEGG analysis. (*C*) Representative western blot and statistical results of protein levels of p-PI3K, PI3K, p-Akt, Akt, p-P65, and P65 (*n* = 4 per group). *P < 0.05 vs. CTL + AAV9-GFP group. #*P* < 0.05 vs. ISO + AAV9-GFP group. KEGG, Kyoto Encyclopedia of Genes and Genomes.

Furthermore, KEGG analysis and GSEA were also performed to screen the candidate signalling pathways involved in ISO-induced VAs. KEGG analysis and GSEA showed that intersecting DEGs were mainly associated with inflammatory response-related signalling pathways, such as the cytokine–cytokine receptor interaction, Phosphatidylinositol 3-kinase (PI3K)/Protein Kinase B (Akt) signalling, Nuclear factor kappa-B (NF-κB) signalling, Tumor necrosis factor (TNF) signalling (*Figure* *7A* and *B*). Among these inflammatory response-related signalling pathways, we focused on PI3K/Akt signalling and NF-κB signalling. Then the western blot analysis further confirmed the KEGG and GSEA findings, PI3K/Akt signalling was activated following ISO treatment and this signalling was inactivated after AAV9-TNIP3 injection (*Figure 7C*). Moreover, NF-κB signalling was activated by ISO treatment, and AAV9-TNIP3 injection significantly inhibited NF-κB signalling (*Figure 7C*). These findings indicated that PI3K/Akt/NF-κB signalling is responsible for the protective effect of TNIP3 overexpression on ISO-induced HF.

### PI3K/Akt signalling activation offset the protective effect of tumour necrosis factor alpha-induced protein 3-interacting protein 3 overexpression on isoproterenol-induced cardiac inflammation and ventricular arrhythmias

**Figure 8:**
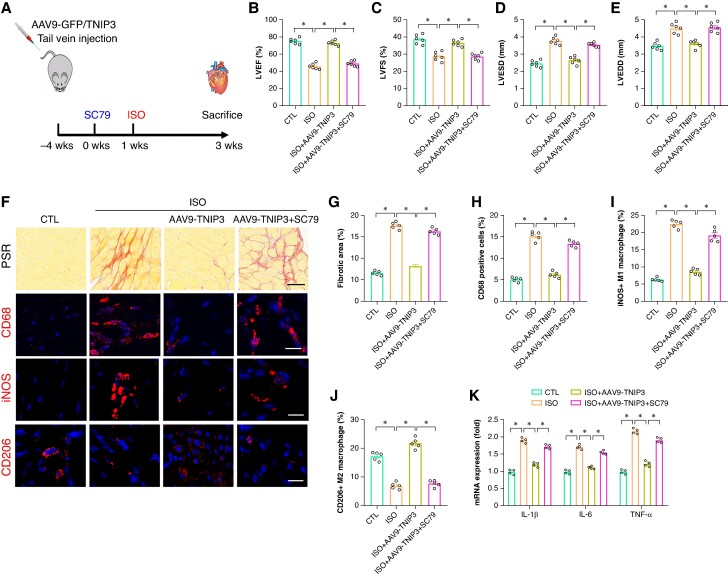
PI3K/Akt signalling activation offset the protective effect of TNIP3 overexpression on ISO-induced cardiac inflammation. (*A*) Schematic diagram of experimental procedure *in vivo* study. (*B–C*) Quantitative of LVEF and LVFS (*n* = 6). (*D–E*) Quantitative of LVESD and LVEDD (*n* = 6). (*F*) Representative images of PSR-stained and CD68, iNOS, and CD206-stained heart sections and (*G*) quantitative data of percentage of the fibrotic area (*n* = 5 per group) and (*H–J*) quantitative results of CD68, iNOS, and CD206-positive cells (*n* = 5 per group). (*K*) Statistical results of mRNA levels of *IL-1β*, *IL-6*, and *TNF-α* (*n* = 4 per group). *P < 0.05. iNOS, inducible nitric oxide synthase.

To further verify whether PI3K/Akt signalling contributes to the protective effect of TNIP3 overexpression on ISO-induced cardiac remodelling. SC79, a specific activator of PI3K, was administered 0.5 h before the ISO treatment *in vivo* (*Figure 8A*). SC79 treatment significantly abolished the protective effect of TNIP3 overexpression on ISO-induced cardiac dysfunction and cardiac enlargement (*Figure 8B–E*). Meanwhile, SC79 treatment also significantly offset the protective effect of TNIP3 overexpression on ISO-induced cardiac fibrosis (*Figure* *8F* and *G*) and proinflammatory response by promoting M1 macrophage polarization (*Figure 8F, H–K*).

**Figure 9:**
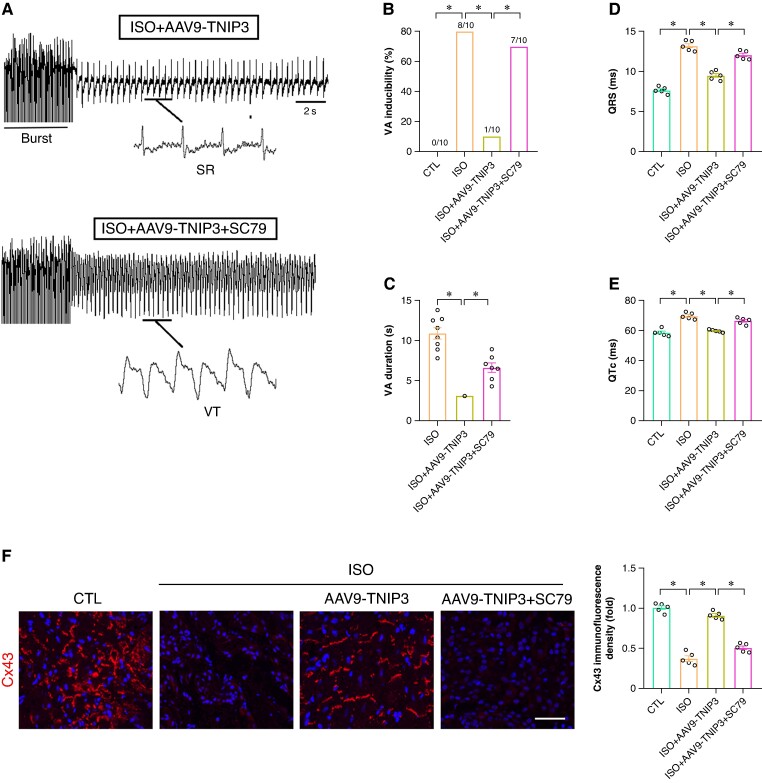
PI3K/Akt signalling activation offset the protective effect of TNIP3 overexpression on ISO-induced VA. (*A*) Representative examples of VA induced by burst-stimulating. (*B*) Ratio of burst-induced VA and (*C*) duration of VA (*n* = 10 per group). (*D–E*) Quantitative of QRS interval and QTc (*n* = 5 per group). (*F*) Representative western blot and statistical results of Cx43 protein expression (*n* = 5). *P < 0.05. SR, sinus rhythm; VA, ventricular arrhythmia.

In addition, PI3 K agonist SC79 was also used to check the effect of PI3K/Akt signalling in ISO-induced electrical remodelling and VAs. Burst pacing revealed that SC79 treatment counteracted the VAs inhibition effect of TNIP3 overexpression (*Figure 9A–C*). Meanwhile, SC79 administered significantly increased QRS duration and QTc compared to ISO + AAV9-TNIP3 group. In terms of Cx43 expression, SC79 treatment notably decreased Cx43 immunofluorescence density in the ventricle compared to ISO + AAV9-TNIP3 group (*Figure 9F*). These data further revealed that PI3K/Akt signalling activation offset the protective effect of TNIP3 overexpression on ISO-induced cardiac inflammation and VAs.

## Discussion

**Figure 10:**
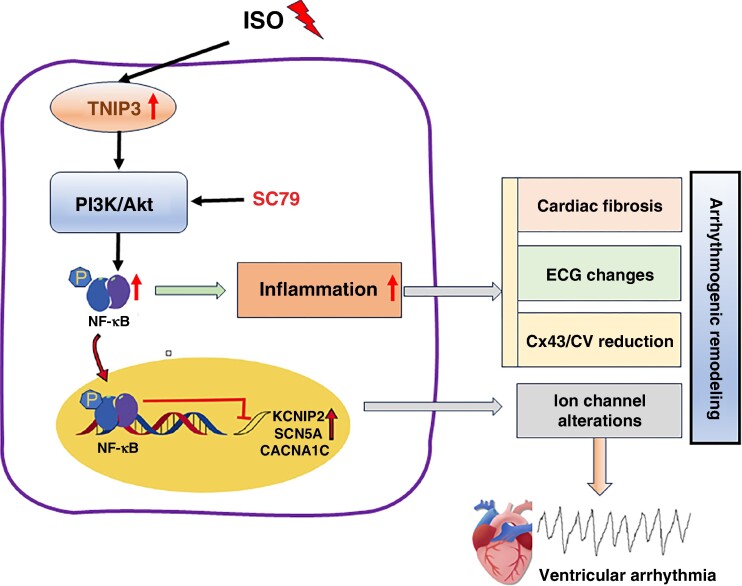
Schematic diagram illustrating that TNIP3 overexpression inhibited cardiac remodelling induced by ISO by attenuating inflammation via the PI3K/Akt/NF-κB signalling pathway. ISO increases TNIP3 expression, which activates PI3K/Akt signalling and promotes NF-κB signalling pathway activation, thus promoting inflammatory response, which leads to arrhythmogenic remodelling and eventually ventricular arrhythmia.

In this study, we uncovered the potential role of TNIP3 in the failing heart and the underlying mechanisms. We observed that ISO treatment significantly up-regulated TNIP3 expression in the hearts of HF models. And TNIP3 overexpression effectively decreased the incidence of VAs. Additionally, overexpression of TNIP3 significantly improved cardiac function, inhibited myocardial fibrosis, and suppressed proinflammatory response by promoting M1 to M2 macrophage polarization. Overexpression of TNIP3 also significantly reversed abnormalities in cardiac ion channel protein expression. Mechanistically, PI3K/Akt/NF-κB signalling is responsible for the protective effect of TNIP3 overexpression on ISO-induced HF. And PI3K/Akt signalling inhibition offset the protective effect of TNIP3 overexpression on ISO-induced cardiac inflammation and VAs (*Figure 10*).

HF is one of the leading causes of morbidity and mortality worldwide. In patients with HF, about half of sudden and unexpected deaths are caused by VAs.^21^ Some mechanisms are mainly related to the overactivation of the sympathetic nervous system and increased catecholamine levels, which continuously stimulate the β-adrenergic receptors (β-AR), thereby promoting cardiac remodelling and arrhythmias during HF.^22^ In basic experimental research, ISO as a β-AR agonist, is widely used to simulate the adverse effects of sustained β-AR activation.^23^ This study found that ISO-induced HF in mice significantly prolonged QTc, electrophysiological examination revealed a significant increase in the incidence of VAs, and a decrease in ventricular CV. Previous studies have also observed similar phenomena, with ISO-induced prolongation of the QTc interval and increased susceptibility of mice to VAs.^24,25^ Additionally, this study found significant upregulation of TNIP3 in the ventricles of HF mice treated with ISO, and that upregulation of TNIP3 significantly reduced the susceptibility to VAs. Previous research indicates that TNIP3 is a negative regulator of myocardial hypertrophy, significantly up-regulated in neonatal rat cardiomyocytes stimulated by phenylephrine and in mouse hearts undergoing TAC surgery.^16^ Based on this, we believe that TNIP3 may play a crucial role in the occurrence and development of VAs following HF.

Cardiac structural remodelling, including ventricular inflammation and fibrosis, forms a crucial basis for the occurrence of VAs.^26^ Previous studies have shown that suppressing inflammation and fibrosis significantly reduces susceptibility to VAs after HF, while activating inflammation and exacerbating fibrosis promotes the occurrence of arrhythmias.^27,28^ Additionally, studies have found that overexpression of TNIP3 provides some protection against hepatic ischaemia-reperfusion injury.^15^ The absence of TNIP3 worsens cardiac function and exacerbates remodelling after pathological myocardial hypertrophy induced by TAC; however, cardiac-specific overexpression of TNIP3 can mitigate pathological myocardial hypertrophy.^16^ This study further found that overexpression of TNIP3 improved cardiac function and fibrosis and alleviated the inflammatory response in ISO-induced HF. Therefore, overexpression of TNIP3 can inhibit post-HF cardiac structural remodelling, thereby reducing the occurrence of VAs.

Cardiac electrical remodelling is another important substrate for the occurrence of VAs after HF.^8^ In the state of HF, the electrical properties of ventricular cardiomyocytes undergo complex changes, which easily trigger VAs. These electrophysiological changes include prolonged action potential duration (APD) and reduced CV due to changes in repolarization. At the cellular and molecular levels, these changes involve various factors, including ion channels and intercellular gap junctions. The prolongation of APD and significant delay in repolarization are closely related, which may increase the susceptibility to malignant arrhythmias through mechanisms such as triggered activity or re-entry.^29^ Cx43 is the main connexin found in ventricular gap junctions, and its deficiency is associated with slow and dispersed conduction.^30,31^ This study found that in ISO-induced HF mice, the protein expression of Cav1.2, Kv1.5, and Kv4.2 were significantly down-regulated, while overexpression of TNIP3 could reverse these adverse changes. Moreover, a recent study also suggests that the expression of Kv1.5, Kv4.2, Cav1.2, and Cx43 proteins was also down-regulated in ISO-induced HF mice. The increase in Cav1.2 levels observed in our study suggests a potential increase in calcium currents, which could indeed be associated with longer APD and QT intervals under typical circumstances. However, our findings also indicate modulatory effects on repolarizing potassium channels, which could counterbalance the effects of increased Cav1.2 expression. Increased potassium currents can facilitate more rapid repolarization, potentially offsetting the prolongation of APD and QT length that would otherwise result from enhanced calcium influx. The balance between calcium influx and potassium efflux is crucial for maintaining cardiac stability and preventing arrhythmogenic risks. By enhancing potassium channel activity, the effects of increased Cav1.2 on APD and QT length may be mitigated, aligning with the protective roles observed. Therefore, we believe that overexpression of TNIP3 can inhibit ventricular electrical remodelling after HF, thereby reducing the occurrence of VAs.

TNIP3 was initially identified in human monocyte-like macrophages during infection with Listeria monocytogenes. It has been demonstrated that TNIP3 negatively regulates NF-κB activation in response to LPS,^32^ and is also implicated in inflammatory bowel disease.^12^ We employed RNA-Seq of cardiac tissue from ISO-infused mice to explore the signalling networks related to ISO-induced cardiac dysregulation and TNIP3-mediated cardio-protection. Our findings indicated that the PI3K/AKT axis was one of the most significantly enriched networks. The PI3K/AKT axis critically modulates normal physiological processes, including cell survival, proliferation, growth, apoptosis, and angiogenesis.^33^ Modulation of the PI3K/Akt pathway has been shown to mitigate myocardial fibrosis and remodelling caused by diabetes, isoproterenol, and other factors.^34^ Chronic activation of AKT is deleterious, with prolonged overexpression of AKT in HF leading to cardiac fibrosis and pathological hypertrophy. The PI3K/AKT pathway is upstream of NF-κB, and AKT activation prompts the translocation of NF-κB to the nucleus, where it acts as an inflammatory factor in cardiac tissue.^35,36^ Inhibiting the AKT/NF-κB pathway mitigates ISO-induced cardiac remodelling.^37^ Additionally, recent studies have shown that blocking the NF-κB pathway can reduce the susceptibility to VAs following HF.^24,38^ In our study, the PI3K/Akt/NF-κB signalling pathway is identified as responsible for the protective effects of TNIP3 overexpression in ISO-induced HF. Furthermore, inhibition of PI3K/Akt/NF-κB signalling negates the protective effects of TNIP3 overexpression against ISO-induced cardiac inflammation and VAs. Consequently, we propose that TNIP3 may regulate the occurrence and progression of VAs post-HF by modulating PI3K/Akt/NF-κB signalling.

In addition, our results indicate that TNIP3 levels are already elevated in failing hearts. However, a further increase can ameliorate electrical disturbances, such as QRS and QT prolongation and alterations in ion channel expression. This suggests that the elevation of TNIP3 may represent a compensatory mechanism of the heart. The natural increase of TNIP3 in failing hearts appears to be an intrinsic effort by the heart to counteract stress and pathological signalling, thus serving as a compensatory response. We propose that although endogenous TNIP3 levels rise in response to cardiac stress, these levels might still be insufficient to fully counteract the deleterious effects of HF, including electrical disturbances. Our observations suggest that augmenting TNIP3 beyond its naturally elevated levels could provide the additional threshold necessary to effectively mitigate electrical abnormalities, indicating that while TNIP3 is part of the cardiac response to dysfunction, its full protective potential is only realized when significantly overexpressed. Finally, we explore the mechanistic basis of how additional TNIP3 overexpression could rectify abnormalities such as QRS and QT prolongation and changes in ion channel expression. This includes potential effects on calcium handling, modulation of inflammatory pathways, and both direct and indirect effects on ion channel gene expression. Given that the specific mechanisms underlying the role of TNIP3 in cardiac electrophysiology and its interactions with other molecular pathways in HF are not yet fully understood, future research will aim to further elucidate these aspects.

### Limitations

Of course, there are still some questions that need to be addressed. Firstly, it should be noted that our results do not provide direct evidence for a mechanistic or causative link between TNIP3 and VAs, which remains to be elucidated in future studies. Secondly, since we used systemic TNIP3 overexpression mice rather than cardiac-specific overexpression mice, we cannot rule out the possibility that TNIP3 and PI3K/Akt/NF-κB signalling in other cell types within the heart may contribute to the reduced susceptibility to VAs in the context of HF. Additionally, clinical and experimental evidence suggests complex pathophysiological mechanisms underlying VAs, including electrical remodelling, structural remodelling, autonomic nervous system changes, and Ca^2+^ handling abnormalities. In the current study, we primarily discussed the regulation of electrical and structural remodelling by TNIP3 in HF mice, but the relationship between TNIP3 and Ca^2+^ handling abnormalities or the autonomic nervous system was not investigated. Fourth, TNIP3 expression was up-regulated in the cardiac tissue of HF models, and overexpression of TNIP3 protected cardiac function. The underlying reason for this may be a compensatory response aimed at counteracting the harmful pathological changes that occur during HF, thereby preserving cardiac function. Therefore, overexpression of TNIP3 can further enhance its protective role, helping to slow down or reverse the progression of HF. Finally, extrapolating data obtained from genetically modified mice to humans is always challenging. Therefore, to support our conclusions regarding the translational application of TNIP3 regulators, further experiments on human samples should be considered in future studies.

## Conclusions

This study is the first to provide evidence that TNIP3 overexpression reduces the susceptibility of failing hearts to VAs by inhibiting cardiac remodelling via the PI3K/Akt/NF-κB signalling pathway. TNIP3 represents a novel therapeutic target for the treatment of heart failure-related VAs.

## Supplementary Material

euaf002_Supplementary_Data

## Data Availability

The data underlying this article will be shared on reasonable request to the corresponding author.
