# Supplementary material for: Tumour necrosis factor alpha-induced protein 3-interacting protein 3 overexpression protects against arrhythmogenic remodelling in the heart failure mice
Source: Europace. 2025 Jan 13;27(1):euaf002. doi: 10.1093/europace/euaf002 (PMC11757166; doi:10.1093/europace/euaf002)
Supplement: euaf002_Supplementary_Data [file euaf002_supplementary_data.docx]

**Supplementary Material**

**1 Supplementary Methods**

**1.1 Echocardiography and hemodynamics**

Echocardiography analysis was performed as previously reported [1]. In brief, echocardiography was performed under continuous anesthesia with 1.5% to 2% isoflurane, using a Mylab30CV (ESAOTE) ultrasound system with a 15Mz probe. Cardiac measurements included examination of left ventricular end-diastolic diameter (LVEDD), left ventricular end-systolic diameter (LVESD), left ventricular ejection fraction (LVEF) and left ventricular fractional shortening (LVFS).

**1.2 Histological analysis**

Embedded heart tissues were cut into 5μm sections. Picric-Sirius red (PSR) staining to analyze the extent of myocardial fibrosis according to the manufacturers’ instructions. Cardiac morphology and the extent of cardiac fibrosis and hypertrophy were assessed using Image J software (NIH Image, Bethesda, MD, USA).

**1.3 Immunohistochemistry (IHC)**

The mouse heart tissue sections were subjected to antigen retrieval and subsequently washed in 3% H_2_O_2_ at room temperature for 20 minutes. Following this, the sections were blocked with 10% bovine serum albumin (BSA) at 37°C for 30 minutes. The TNIP3 primary antibody (BT-AP15046, 1:100 dilution, BT LAB) was then applied and the sections were incubated at 4°C overnight. Subsequently, the sections were washed with PBS and treated with the Goat anti-Rabbit Detection Kit (BLRE006-200T, Biolight) at 37°C for 1 hour. Positive signals on the slices were visualized using diaminobenzidine working solution (ZLI-9018, ZSGB-BIO) after washing with PBS. Hematoxylin staining (G1004, Servicebio) was performed, followed by washing with ddH_2_O. Finally, the slices were sealed with a resin sealant (BA-7004, Baso), and images were obtained using Digital Pathology Slide Scanners (Aperio Versa 200, Leica).

**1.4 Immunofluorescence staining**

The paraffin-embedded sections were deparaffinized and retrieved for antigen. Then, sections were stained with antibody against F4/80 (Bioss, China) or inducible nitric oxide synthase (iNOS, ab283655, Abcam) or CD206 (AG2660, Servicebio Technology, China) following fluorescence-labeled secondary antibodies. Each sample's infiltrating macrophage count was determined. Moreover, slides were stained with primary antibodies against connexin 43 (Cx43, #ab235282, Abcam). Nuclei were stained with 4’6-diamidino-2-phenylindole (Sigma-Aldrich). An OLYMPUS DX51 fluorescent microscope (Tokyo, Japan) was used to take the photos. Each heart-stained sections were examined and captured on camera using a microscopy (×200) with 20 randomly selected fields of vision.

**1.5 Electrocardiogram**

After the HF model, the mice were anesthetized using pentobarbital sodium (40 mg/kg, i.p. Sigma). Standard surface ECGs were consistently recorded using a computer-based Lab System (LEAD7000; Jinjiang Ltd) throughout the experiment. Then the basic parameters were measured and analyzed: duration of QRS, and QTc intervals.

**1.6 Ventricular arrhythmia (VAs) inducibility**

Mice were anaesthetized (pentobarbital sodium, 1%) and then incubated and ventilated with a volume-constant rodent ventilator, and a left thoracotomy was performed. Then, opening the chest along the subcostal margin to fully expose the heart to prepare for the electrophysiological study. The platinum stimulation electrode was placed on the right ventricle. A self-made single-phase action potential electrode was used to record the left ventricle action potential.

According to our previous study, burst pacing protocols were conducted to determine susceptibility to VAs [1]. Briefly, VAs was induced through the last 2s burst pacing, which was repeated three times. VAs was defined as consecutive premature ventricular contractions of at least 2 seconds. Susceptibility to VAs was evaluated based on the VAs incidence and the ratio of sustained to non-sustained VAs [1].

**1.7 Optical mapping**

The optical mapping was performed according to previously reported [2]. Briefly, the mice were heparinized firstly to prevent clotting and anesthetized (pentobarbital sodium, 1%). The hearts were removed and washed immediately with oxygenated Tyrode’s solution (in mM: 127 NaCl, 1.54 NaH_2_PO_4_, 20 NaHCO_3_, 1.0 MgCl_2_ 6H_2_O, 4.7 KCl, 1.72 CaCl_2_ and 11.1 glucose in deionized water, adjusts pH to 7.4) at a constant temperature of 37 C. After aortic catheterization, the heart was perfused retrogradely with Tyrode’s solution. (-)-Blebbistatin (10 mmol/L, Selleckchem, Houston, TX, USA) was used to inhibit the cardiac contractility, after which the potentiometric probe RH237 (Invitrogen, Carlsbad, CA) was mixed into the Tyrode’s solution and perfused into the mouse heart for 10 min. The heart was excited by the light source of green LED lamp (530 nm), followed by an electrode was placed at the tip of the left ventricle for stimulation at different frequencies. The fluorescence images were captured with the CMOS camera (MiCAM05 Ultima, SciMedia, California, USA) by 1000 frames per second and 100 × 100 pixels. SciMedia customized software (SciMedia, Costa Mesa, CA) was used to initiate fluorescence signal recording and statistics of cardiac conduction velocity (CV).

**1.8 RNA sequencing (RNA-seq)**

Total RNA was extracted from LV tissues using TRIzol™ Reagent (Invitrogen) and subjected to the BioMarker (Beijing, China) for RNA-seq. Differential expression genes (DEGs) were considered significantly different when the log2FoldChange ≥1 or ≤−1, and the *P* < 0.05. The selected DEGs were cluster analyzed and a heat map was drawn in MultiExperiment Viewer software [18]. All DGEs were analyzed for enrichment in signaling pathways using statistical methods such as Fisher's exact test and were annotated into pathways available in the Kyoto Encyclopedia of Genes and Genomes (KEGG) database. Signaling pathways with a *P* value < 0.05 were considered statistically enriched. For each Gene Ontology (GO) biological process term, the genes involved were defined as a gene set. The gene sets were then analyzed using gene set enrichment analysis (GSEA) on the Java GSEA platform. Statistical significance was determined based on a *P* value < 0.05 and a false discovery rate (FDR) value < 0.25.

**1.9 Cell culture**

H9c2 cells were obtained from the Cell Bank of the Chinese Academy of Sciences (Shanghai, China) and cultured in Dulbecco’s modified Eagle’s medium with fetal bovine serum (10%), streptomycin (1%), and penicillin. The culture conditions contained a humidified atmosphere (95% air and 5% CO2 at 37°C). The H9c2 cells were incubated with ISO (10 µM) for 12 h.

**1.10 RT-qPCR**

Total RNA was extracted from cardiac tissues or cells using Trizol reagent (Invitrogen). First-strand cDNA was synthesized from total RNA using Prime Script™ RT Master Mix (Takara, Tokyo, Japan). RT-qPCR was performed in a 25μL reaction on the CFX96 Real-Time PCR Detection System (Bio-Rad Laboratories), including 0.4μmol/L primers, 50 ng of cDNA, and 12.5μL TB Green Premix Ex Taq II (Takara). The expression levels of target genes were normalized to the expression levels of beta-actin, which was considered as an endogenous internal control. The primer sequences were as exhibited in **Table S1**.

**1.11 Western blotting**

Western blotting was performed to evaluate protein expression levels as described previously [1]. This manuscript's primary antibodies are: TNIP3 (1:500, NBP1-77365, NOVUS), Cx43 (1:2000, ab117843, Abcam), IL-1β (1:1000, AF5103, Affbiotech), IL-6 (1:500, sc-57315, Santa), TNF-α (1:1000, 17590-1-AP, Proteintech), p-P65 (1:1000, 3033, CST), T-P65 (1:3000, 8242, CST), p-PI3K (1:500, ab182651, Abcam), T-PI3K (1:2000, 4257, CST), p-Akt (1:1000, 4060, CST), T-Akt (1:2000, 9272, CST), Cav1.2 (1:500, ab84814, Abcam), Kv1.5 (1:2000, 21659-1-AP, Proteintech), Kv4.3 (1:1000, DF13239, Affbiotech) and GAPDH (1:10000, ab181602, Abcam). The total protein levels were normalized to GAPDH.

**2 Supplementary Tables**

**Table S1**. Mouse Primers for RT-PCR

| Gene | Forward Primers | Reverse Primers |
| --- | --- | --- |
| *Collagen-I* | CCTGGCAAAGACGGACTCAAC | GCTGAAGTCATAACCGCCACTG |
| *Collagen-III* | CTG​TAA​CAT​GGA​AAC​TGG​GGA​AA | CCA​TAG​CTG​AAC​TGA​AAA​CCA​CC |
| *TGF-β* | CCA​GAT​CCT​GTC​CAA​ACT​AAG​G | CTC​TTT​AGC​ATA​GTA​GTC​CGC​T |
| *IL-1β* | TGCCACCTTTTGACAGTGAT | TGTGCTGCTGCGAGATTTGA |
| *IL-6* | TACCAGTTGCCTTCTTGGGACTGA | TAAGCCTCCGACTTGTGAAGTGGT |
| *TNF-α* | TCTCATGCACCACCATCAAGGACT | ACCACTCTCCCTTTGCAGAACTCA |
| *iNOS* | CACCACCCTCCTCGTTC | CAATCCACAACTCGCTCC |
| *Arg1* | GGAAGACAGCAGAGGAGGTG | TCAGTCCCTGGCTTATGGTT |
| *Mrc1* | AGTGATGGTTCTCCCGTTTC | TGGGCTCAGGTAGTAGTGTTTT |
| *Gapdh* | CATCTTCTTGTGCAGTGCC | CAAATCCGTTCACACCGAC |

**References**

1. Yang HJ, Kong B, Shuai W, Zhang JJ, Huang H. Shensong Yangxin attenuates metabolic syndrome-induced atrial fibrillation via inhibition of ferroportin-mediated intracellular iron overload. Phytomedicine. 2022;101:154086.
2. Liu Y, Li J, Xu N, Yu H, Gong L, Li Q, Yang Z, Li S, Yang J, Huang D, Xue Y, Xue G, Liu J, Chen H, Zhang R, Li A, Zhao Y, Li P, Li M, Liu M, Wang N, Cai B. Transcription factor Meis1 act as a new regulator of ischemic arrhythmias in mice. J Adv Res. 2022;39:275-289.
